# The Effect of Prebiotics and Oral Anti-Diabetic Agents on Gut Microbiome in Patients with Type 2 Diabetes: A Systematic Review and Network Meta-Analysis of Randomised Controlled Trials

**DOI:** 10.3390/nu14235139

**Published:** 2022-12-02

**Authors:** Omorogieva Ojo, Xiaohua Wang, Osarhumwese Osaretin Ojo, Joanne Brooke, Yiqing Jiang, Qingqing Dong, Trevor Thompson

**Affiliations:** 1 School of Health Sciences, Avery Hill Campus, University of Greenwich, London SE9 2UG, UK; 2 The School of Nursing, Soochow University, Suzhou 215006, China; 3 Smoking Cessation Department, University Hospital, London SE13 6LH, UK; 4 Faculty of Health, Education and Life Sciences, Birmingham City University, Birmingham B15 3TN, UK; 5 School of Human Sciences, Avery Hill Campus, University of Greenwich, London SE9 2UG, UK

**Keywords:** prebiotics, oral anti-diabetic agents, gut microbiome, glycated haemoglobin, type 2 diabetes, Network meta-analysis, meta-analysis

## Abstract

Background: Nutritional interventions such as the use of prebiotics can promote eubiosis of gut microbiome and maintain glucose homeostasis in patients with type 2 diabetes (T2D). However, it would appear that results of the effects of prebiotics on the community of microbes in the gut are not consistent. Aim: To examine the effect of prebiotics and oral antidiabetic agents on gut microbiome in patients with T2D. Methods: The PRISMA Extension Statement for Systematic Reviews and Network Meta-analyses was used to conduct this review. Searches were carried out in EMBASE, EBSCO-host databases, Google Scholar and the reference lists of articles for studies that are relevant to the research question, from database inception to 15 August 2022. The search strategy was based on PICOS framework. Network Meta-analysis which allows the estimation of relative treatment effects by combing both direct trial evidence (e.g., treatment A vs. treatment B) and indirect evidence was conducted. Furthermore, pairwise meta-analysis was also carried out to estimate effect sizes based on head-to-head comparisons of treatments and/or control conditions. Results: Findings of the Network meta-analysis revealed that prebiotics significantly reduced HbA1c compared with control and the SMD was −0.43 [95% CI, −0.77, −0.08; *p* = 0.02], whereas there was no significant difference (*p* > 0.05) between the other treatments and control. In addition, anti-diabetic agents including glipizide and metformin also reduced HbA1C, although these were not significantly different (*p* > 0.05) from control. While prebiotics promoted *Bifidobacterium* and *Akkermansia*, the improvements were not significantly different (*p* > 0.05) from control. On the other hand, metformin decreased the relative abundance of *Bifidobacterium*, but increased *Lactobacillus* and *Akkermansia*, although the differences were not significant (*p* > 0.05) compared with control. With respect to fasting blood glucose and BMI, the effects of prebiotics and oral antidiabetic agents did not differ significantly (*p* > 0.05) from controls. Conclusions: The findings of the systematic review and Network meta-analysis demonstrated prebiotics were significantly (*p* < 0.05) more effective in reducing HbA1c than control in patients with T2D. However, the effects of prebiotics and oral antidiabetic agents did not differ significantly (*p* > 0.05) from the controls in relation to fasting blood glucose, post-prandial blood glucose, body mass index and the genera of gut bacteria examined. More studies are required to fully investigate the effects of prebiotics and oral antidiabetic agents in patients with T2D

## 1. Introduction

The prevalence of type 2 diabetes (T2D) is increasing globally. It is estimated that by 2040, approximately 642 million people will have the condition worldwide [1]. Genetic predisposition and lifestyle factors such as lack of physical activities and poor nutritional intake which can lead to overweight and obesity are reported to be involved in the etiology of T2D [2]. Furthermore, diets with low fibre and high saturated fats and sugar, such as Western diets, may also influence gut microbial diversity and cause reduction in specific bacteria taxa and imbalance in gut microbiome [3,4].

There is evidence from meta-analysis of randomised controlled trials (RCTs) that T2D is associated with disequilibrium of gut microbial community and gut microbiota dysbiosis is implicated in the pathogenesis of type 2 diabetes [5]. Therefore, nutritional interventions including prebiotics have been used to promote eubiosis of gut microbiome and maintain glucose homeostasis in patients with T2D [6,7]. In addition, the role of oral anti-diabetic agents in modulating dysbiosis of gut microbiome may be a possible pathway by which these drugs regulate glucose balance [8,9].

### 1.1. Description of the Intervention

The concept of prebiotics relates to the food component that is not digestible. Dietary prebiotics must be resistant to gastric acid and should not be hydrolised by the effect of mammalian enzyme, and should be resistant to intestinal absorption. It should also be beneficial to the hosts through selective promotion of the growth of bacteria in the colon, not causing negative effects to the hosts including not stimulating the growth of pathogenic microorganisms [10]. Prebiotics have also been recently defined as substrates (non-viable) which are used selectively by the host microorganisms which leads to benefits [10].

The definition of prebiotics has been revised to include ingredients that are selectively fermented and allows changes that are specific to the community and actions of microbes inhabiting the gastrointestinal tract which confers effect on the host which are beneficial physiologically [11]. Prebiotics are different from most dietary fibres including pectins, cellulose and xylans which promote the development of a broad variety of gut microbes [12].

Although prebiotics are not the only substrates that can affect the gut microbial community, a primary criterion that distinguishes prebiotics from other substrates is their selective utilisation by host microorganisms [12]. While a selective effect does not mean utilisation by just one microbial group, it may include several microbial groups, but not all the microbial groups [12].

Metformin is one of the oral anti-diabetic agents and it is a biguanide [13]. It is a first line medication for treating T2D and is effective in lowering body weight and cardiovascular risks [13]. Other oral anti-diabetic agents that are associated with modulation of gut microbiota include sulfonylurea and acarbose [9,14].

### 1.2. How This Intervention Might Work

Dysbiosis of intestinal microflora has been shown to have significant effect in the pathogenesis of metabolic disorders such as T2D [15]. Therefore, sustaining an ecosystem that is healthy and having good lifestyle and feeding habits are useful approaches in managing T2D [15]. The consumption of prebiotics may regulate gut microbiota dysbiosis and enable the growth of beneficial microbes [11]. There is evidence to suggest that prebiotic dietary fibre is a selective substrate that is utilised by bacteria which are beneficial to the host including *Bifidobacterium* and *Lactobacillus* that promote the health of the host [10]. For example, prebiotics may promote the growth of bacteria that are beneficial including *Lactobacillus*, *Bifidobacterium*, *Akkermansia*, *Eubacterium* and *Roseburia* [10].

Prebiotics are usually metabolised by the gut microbes through a process of fermentation to produce metabolites which are useful to the host [10]. The end product of metabolism of prebiotics is short chain fatty acids (SCFAs), which are primarily propionic, butyric and acetic acid [10]. SCFAs influence the integrity of the gut epithelium, immunity, glucose homeostasis, lipid profile and body weight [11]. In addition, SCFAs have effects on insulin resistance, suppress appetite and lipolysis, increase expenditure of energy and promote insulin sensitivity and production [9,15].

While butyrate is a good source of energy for colonocytes and enterocytes, propionate is a substrate for intestinal and hepatic gluconeogenesis, and the most abundant SCFA found in circulation is acetate [11]. The phenomenon of cross-feeding by other bacteria has also been discussed as possible mechanism employed in the production of SCFAs which are crucial for the intestinal health and other health benefits in areas distant to the gut [12,16]. Cross feeding is a process where a substrate stimulates the growth of members of the gut microbiota which produces metabolites which are utilised by other microbes to produce butyrate and other SCFAs [12].

Antidiabetic agents have also been shown to restore the richness and diversity of the gut microbial community to some level and have demonstrated ability to promote the growth of some useful bacteria [15]. In particular, anti-diabetic agents not only influence gut microbiota, in turn, microbiota affects how the individual responds to those drugs which explains the bidirectional relationship between microbes in the gut and anti-diabetic medications [17]. Metformin has been shown to reduce blood glucose in patients with T2D by interacting with microbes in the gut including altering the composition and diversity of gut microbiome [8,18].

### 1.3. Why It Is Important to Do This Review

The definition of prebiotics has been evolving over the years, therefore, a good knowledge of their effect on gut microbiome in patients with T2D will help in enriching our understanding of this concept, broaden their application and health related outcomes [10,11,12,16]. Furthermore, an understanding of gut microbial ecology in patients with T2D is useful in developing effective approaches to regulate gut microbiota dysbiosis for purposes that are preventive and therapeutic [15]. It has been suggested that the effectiveness of prebiotics in patients with T2D is based on the modulation of gut microbiome although the results are not consistent [19]. In addition, it seems the systematic reviews and/or meta-analysis conducted previously [20,21,22] have not focused on the effect of prebiotics on gut microbiome in patients with T2D. In other systematic reviews, studies involving probiotics [23] and prebiotics or symbiotics supplementations [24,25] were included. In addition, the review by Merkevicius et al. [24] included one animal study, but did not involve meta-analysis. The Bock et al. [25] review included patients with type 1 diabetes. In our previous systematic review [26], we examined the effect of dietary fibre in regulating the imbalance in the gut microbial community, but did not compare this with oral antidiabetic agents. In contrast, the current review is a systematic review and Network Meta-analysis (NMA) of RCTs which seeks to evaluate the impact of prebiotics and oral anti-diabetic agents on gut microbiota and metabolic parameters in patients with T2D.

### 1.4. Research Questions

Are prebiotics more effective than a control in managing patients with T2D?

What is the comparative effectiveness of prebiotic treatment or treatment with oral antidiabetic agents in patients with T2D?

Aim.

To examine the effect of prebiotics and oral anti-diabetic agents on gut microbiome in patients with T2D.

## 2. Methods

The Preferred Reporting Items for Systematic Reviews and Meta-Analyses Extension Statement for Reporting of Systematic Reviews Incorporating Network Meta-analyses of Health Care Interventions was used to conduct this systematic review and Network meta-analysis (PRISMA-ES for NMA) [27,28].

Registration: This systematic review and Network meta-analysis protocol was registered with Prospero and the Registration Number was CRD42022352060.

### 2.1. Studies Included

Only RCTs were selected for the review.

### 2.2. Participants of Interest

Patients with T2D were participants included in the review.

### 2.3. Types of Interventions

Pre-biotics and oral anti-diabetic agents were the interventions of choice.

### 2.4. Outcome Measures

The following were the outcomes of interest:

Gut Microbiome: *Lactobacillus*, *Bifidobacterium*, *Ruminococcus*, *Bacteroides*, *Roseburia*, *Clostridium* and *Akkermansia* (Relative abundance and genera only).

Blood Glucose Parameters: glycated haemoglobin (HbA1c), fasting blood glucose (FBG) and postprandial blood glucose.

Body Mass Index.

### 2.5. Search Strategy

EBSCOHost was searched for relevant articles using the Health Sciences Research Databases (which includes MEDLINE, APA PsycArticles, Academic Search Premier, CINAHL Plus with Full Text, Psychology and Behavioral Sciences Collection and APA PsycInfo databases). Furthermore, EMBASE and Google Scholar were additional databases searched. The reference lists of articles were searched for studies that were relevant to the research question. The searches were carried out from database inception to 15 August 2022. The Population, Intervention, Comparator, Outcomes, Studies (PICOS) tool was used to define the research question and establish the search strategy [29]. The search terms included synonyms and medical subject headings and these were combined with Boolean operators (OR/AND) (Table 1). OO and OOO conducted the searches separately and these were cross checked by X.W. and JB. Search results were transferred to EndNote (Analytics, Philadelphia, PA, USA) and duplicates of articles were deleted.

## 3. Collection of Data and Analysis

### 3.1. Study Selection

Criteria for Inclusion: Patients with T2D and those who were 18 years of age or older were selected for the review. Other inclusion criteria were studies involving prebiotics and/or oral antidiabetic agents as interventions and studies that meet the required outcomes, including; gut microbiome, glycaemic parameters and body mass index.

Criteria for Exclusion: Participants younger than 18 years of age, those with gestational diabetes, pre-diabetes and type 1 diabetes, and studies with probiotics and animal models were excluded from the review.

**Figure 1:**
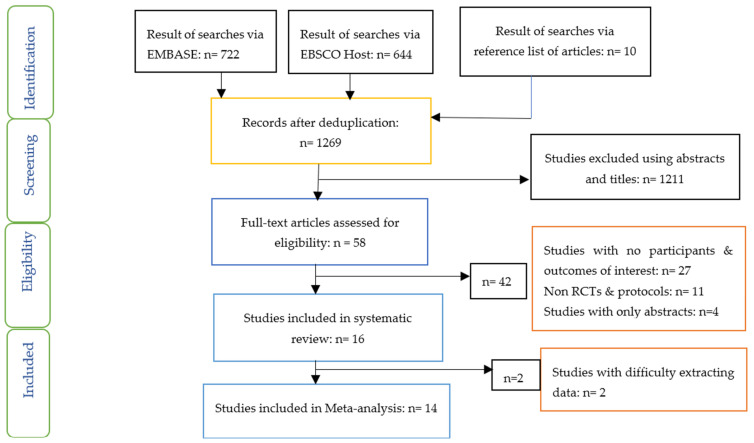
PRISMA flow chart on selection and inclusion of studies.

The PRISMA flow chart (Figure 1) provides details of studies included using the criteria for inclusion and exclusion previously outlined.

### 3.2. Data Extraction and Management

O.O., Y.J., Q.D. and X.W. extracted the data from included articles and these were cross-checked by all authors. Changes from baseline and final values of all parameters of interestwere used for the Network meta-analysis and pairwise meta-analyis [30]. The intervention group data were compared to the control group. Data from studies such as Medina-vera et al. [31] and Pedersen et al. [32] were extracted using the Engauge Digitizer [33]. Furthermore, the units of measurements were converted in some parameters such as fasting blood glucose (mmol/L), glycated haemoglobin (%) and Bifidobacterium (%). Means and standard deviations were calculated from median and 1st–3rd quartiles, respectively, in some parameters.

Risk of Bias Assessment of Studies.

The studies included were evaluated based on the established assessment tool [30]. The domains assessed were attrition bias, selection bias, detection bias, performance bias, reporting bias, and other bias [30]. The Review Manager 5.3 software [34] was used to assess the risk of bias.

## 4. Data Analysis

### 4.1. Network Meta-Analysis (NMA)

NMA was performed within a frequentist framework using the netmeta package [35] in R to compute standardised mean differences (SMDs). NMA allows the estimation of relative treatments effects by combing both direct trial evidence (e.g., treatment A vs. treatment B) and indirect evidence (e.g., in trials where A and B have not been directly compared but have all used a common comparator, e.g., placebo, allowing A and B to be compared indirectly).

Network plots for each outcome were first constructed and examined to identify any intervention comparisons which were disconnected from the main treatment network and which therefore could not be examined using NMA. We then performed NMA and constructed forest plots comparing each treatment to a reference condition (either placebo or inactive control according to what was most commonly employed for that outcome).

A key assumption of NMA is transitivity, which broadly speaking is that trials of different treatment comparisons are broadly similar on important methodological and sample characteristics (such as age, gender etc). If differences do exist that might cause the effect of a treatment to be amplified or diminished in a set of trials regardless of the particular treatment given (e.g., due to use of an older less treatment-responsive sample) then this assumption is violated. We assessed this by inspecting a summary table of key potential effect modifiers of sex, age, etc across the different sets of treatment comparisons. (Refer to table on mean age and sex distribution of treatments in the supplementary file). An alternative method of evaluating inconsistency is by comparing the differences between direct and indirect evidence for each comparison. However, we did not attempt to do this here as the data we examined allowed the computation of SMD exclusively from either direct or indirect evidence but not both.

Pairwise meta-analysis was also conducted to estimate effect sizes based on head-to-head comparisons of treatments and/or control conditions. Pairwise meta-analysis was conducted using Revman.

### 4.2. Meta-Analysis

The Review Manager (RevMan) 5.3 software [34] was used to conduct the meta-analysis. The measure of heterogeneity was the I^2^ statistic [30], and statistical significance of heterogeneity was set at p < 0.10. The fixed effects model was used when heterogeneity was not important (I^2^: 0–40%) and the random effects model was applied when heterogeneity was substantial or considerable (I^2^: 40–100%) [30]. The SMD was used for the meta-analysis.

A subgroup analysis was carried out to examine the effect of prebiotics and oral antidiabetic agents in patients with T2D.

### 4.3. Effect Size

The result of the meta-analysis are depicted as forest plots and in terms of statistical significance, *p* < 0.05 was used to assess the overall effect of the intervention.

## 5. Results

Sixteen studies were included in the systematic review, while fourteen studies were included in the Network meta-analysis (Figure 1). The characteristics of the included studies including countries where studies were conducted, type of study, participants, sample size, mean age, mean diabetes duration, interventions and results/findings are outlined in Table 2. Four studies were carried out in China, three in Italy and two studies in Mexico. One study each was carried out in Japan, Korea, Norway, Canada, Netherlands, UK and Spain. All these studies were randomised controlled studies.

The network plots of the Network meta analysis can be found in the supplementary file (Figure S1 and Table S1).

## 6. Risk of Bias of Studies Included

**Figure 2:**
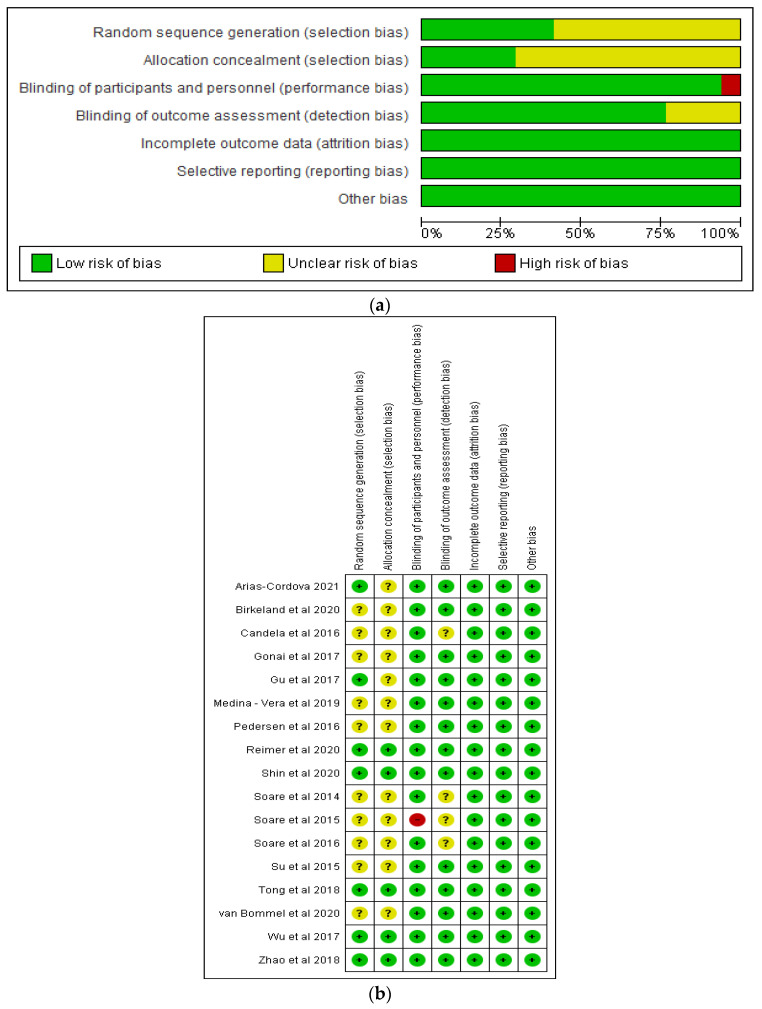
Graphs showing (**a**) risk of bias (**b**) risk of bias summary [18,31,32,36,37,38,39,40,41,42,43,44,45,46,47,48].

Figure 2a,b show the risk of bias graph and risk of bias summary, respectively, of the studies in this review. There was low risk of bias in relation to incomplete outcome data (attrition bias), selective reporting (reporting bias), blinding of participants and personnel, and other bias in all the studies. Nine of the 16 studies demonstrated unclear risk of bias with respect to random sequence generation, while 11 studies demonstrated unclear risk of bias in relation to allocation concealment. In terms of blinding of outcome assessments, there were 3 studies with unclear risk of bias.

## 7. Effects of Interventions

Three distinct areas were identified based on the results of the systematic review and NWM, namely: Gut microbiome; Glycaemic control; and Body Mass Index (BMI).

Gut Microbiome.

The effects of prebiotics and oral antidiabetic agents on gut microbiome were varied (Table 3). For example, Birkeland et al. [37] found significant increase in faecal levels of *Bifidobacteria* following daily supplement of inulin-type fructans, while Gonai et al. [39] observed significant restoration of *Bifidobacteriaceae* in patients with T2D after the consumption of galacto-oligosaccharide. In addition, Zhao et al. [48] found high fibre diet promoted the growth of short chain fatty acid producing microbes in patients with diabetes. However, the effect of prebiotic treatment on *Bifidobacterium*, *Lactobacillus* and *Roseburia* was not significant in Pedersen et al. [32] study.

While Gu et al. [40] and Su et al. [45] found acarbose can increase the relative abundances of *Bifidobacterium* species, Wu et al. [18] noted *Bifidobacterium adolescentis* increased after metformin treatment.

## 8. Bifidobacterium

**Figure 3:**
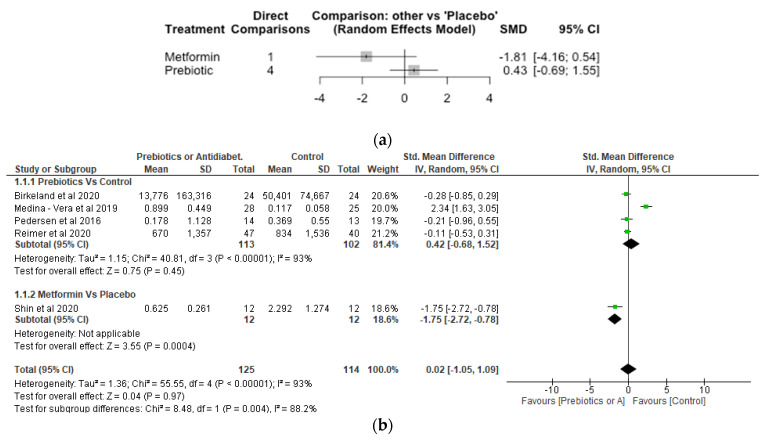
Network Meta-analysis (**a**) and Meta-analysis (**b**) of the effect of treatments versus control on *Bifidobacterium* [31,32,37,41,42].

The Network meta-analysis for *Bifidobacterium* included 5 studies, 239 participants and 3 treatments. The result showed prebiotic treatment increased the relative abundance of *Bifidobacterium* although this was not significantly different compared with placebo with a SMD of 0.43 [95% CI, −0.69, 1.55; *p* = 0.45] (Figure 3a). In contrast, metformin treatment reduced the relative abundance of Bifidobacterium with a SMD of −1.81 [95% CI, −4.16, 0.54; *p* = 0.13] compared to placebo, but again this was not significant. Pairwise meta-analysis conducted to estimate effect sizes based on head-to-head comparisons of treatments and/or control conditions (Figure 3b) found no significant difference (*p* > 0.05) between prebiotic treatment and control on the relative abundance of *Bifidobacterium*. The effect of metformin was significant (*p* < 0.05).

## 9. Lactobaccilus

**Figure 4:**
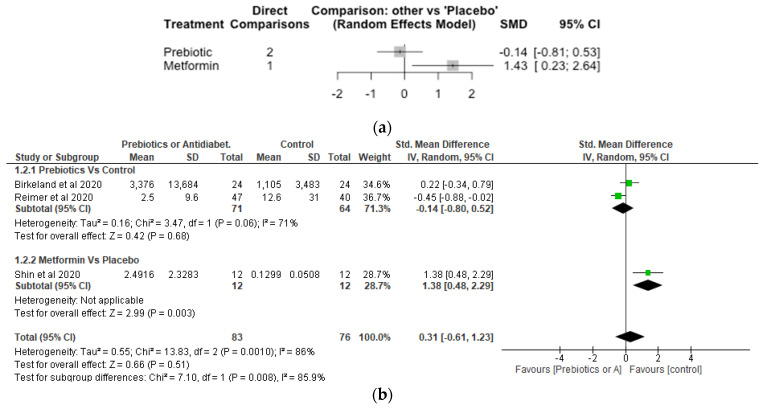
Network Meta-analysis (**a**) and Meta-analysis (**b**) of the effect of treatments versus control on *Lactobaccilus* [37,41,42].

The Network meta-analysis for *Lactobaccilus* included 3 studies, involving 159 participants. The effect of metformin treament on the relative abundance of *Lactobaccilus* showed a significant increase with SMD of 1.43 [95% CI, 0.23, 2.64; *p* = 0.02] compared to placebo (Figure 4a). However, the effect of prebiotic compared to placebo was not significantly different with a SMD of −0.14 [95% CI, −0.81, 0.53; *p* = 0.68]. The meta-analysis (Figure 4b) also showed metformin significantly (*p* < 0.05) increased *Lactobaccilus* compared with control while differences between prebiotics and control did not differ significantly (*p* > 0.05).

## 10. Akkermansia

**Figure 5:**
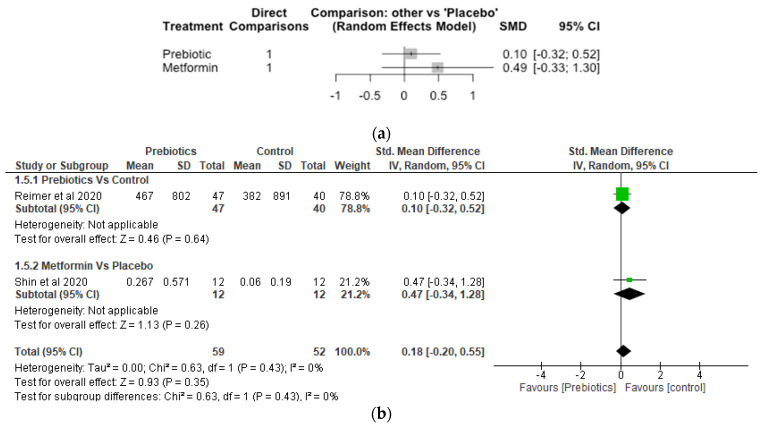
Network Meta-analysis (**a**) and Meta-analysis (**b**) of the effect of treatments versus control on *Akkermansia* [41,42].

The Network meta-analysis for Akkermansia included 2 studies, 111 participants and 3 treatments. Both metformin and prebiotic treatments increased the relative abundance of *Akkermansia*, although the effects did not differ significantly (*p* > 0.05) compared to placebo (Figure 5a). The SMD was 0.10 [95% CI, −0.32, 0.52; *p* = 0.64] for prebiotic and 0.49 [95% CI, −0.33, 1.30; *p* = 0.24] for metformin treatments, respectively, compared with placebo. The results of the meta-analayis did not show any significant difference (*p* > 0.05) between the prebiotic and control, and metformin and control (Figure 5b).

## 11. Glycaemic Control

In the study by Arias-Córdova et al. [36], it was found that the native banana starch (NBS) with a content of 70.5% resistant starch and 10% digestible starch caused a reduction in fasting blood glucose from baseline compared with digestible maize starch with 100% digestible starch content. There was improvement in insulin sensitivity and significant improvement in glycaemic control including significant reduction in parameters such as HbA1c, postprandial blood glucose and fasting blood glucose levels in patients with type 2 diabetes who consumed prebiotic diets compared with control in some studies [31,38,41,43,44,48].

However, following the consumption of prebiotic diets, there was no improvement in glucose control in other studies [32,39].

With respect to the oral antidiabetic agents, Wu et al. [18] found metformin significantly reduced HbA1c and fasting blood glucose levels compared with calorie restricted diet. Furthermore, Tong et al. [46] reported metformin improved Homeostatic Model Assessment for Insulin Resistance (HOMA-IR) compared with control, while Su et al. [45] observed acarbose treatment improved glycemic control in patients with type 2 diabetes. Both dapagliflozin and gliclazide reduced HbA1c and fasting blood glucose levels in the study by van Bommel et al. [47]. Similarly, the acarbose and glipizide groups improved glycemic control, with no significant differences between the two groups [40].

However, Shin et al. [42] reported that *Scutellaria baicalensis* with metformin treatment or placebo did not change the glucose and HbA1c levels.

Glycated Haemoglobin (HbA1c).

**Figure 6:**
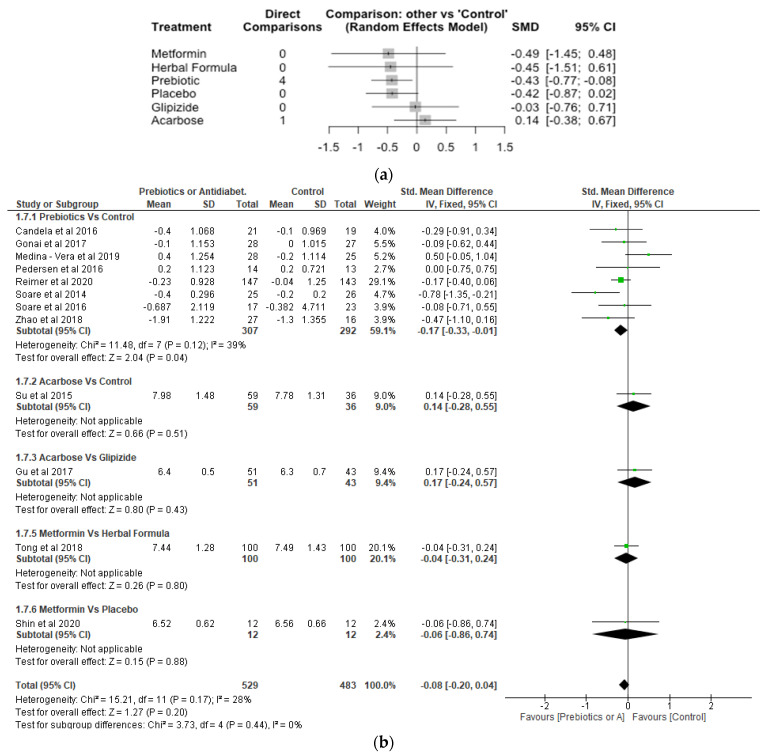
Network Meta-analysis (**a**) and Meta-analysis (**b**) of the effect of treatments versus control on glycated haemoglobin (HbA1c) [31,32,38,39,40,41,42,43,44,45,46,48].

The Network meta-analysis for HbA1c included 12 studies, 7 treatments and 1012 participants (number of observations). Compared with control, glipizide, herbal formula and metformin treatments reduced HbA1c although the difference was not significant (*p* > 0.05). In contrast, prebiotic treatment significantly reduced HbA1c compared to control with a SMD of −0.43 [95% CI, −0.77, −0.08; *p* = 0.02] (Figure 6a). The results of the meta-analysis demonstrated prebiotics significantly (*p* < 0.05) reduced HbA1c compared to control, whereas the differences between the other treatments and control were not significant (*p* > 0.05) (Figure 6b).

Fasting Blood Glucose.

**Figure 7:**
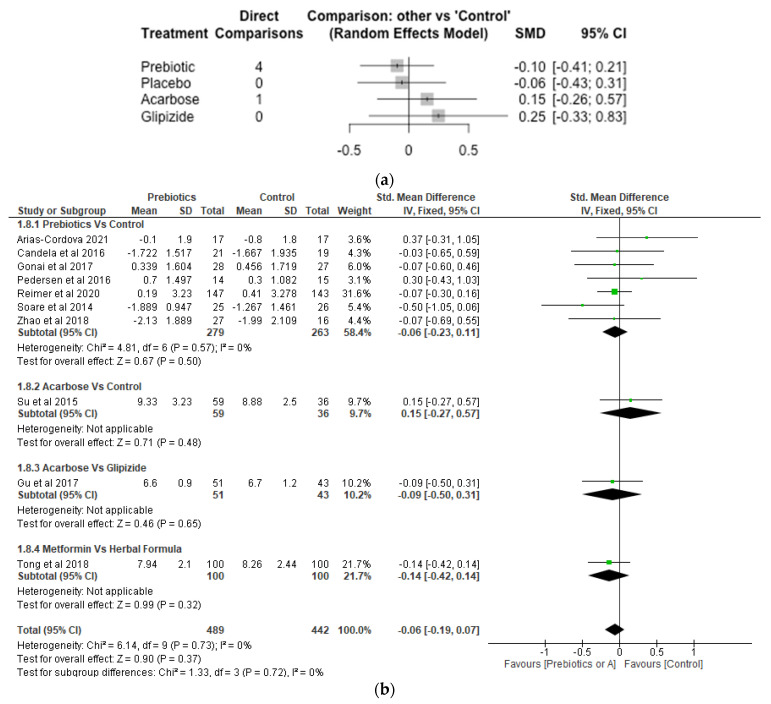
Network Meta-analysis (**a**) and Meta-analysis (**b**) of the effect of treatments versus control on Fasting Blood Glucose [32,36,38,39,40,41,43,45,46,48].

There were 9 studies, 731 participants or number of observations and 5 treatments involved in the Network meta-analysis of fasting blood glucose (Figure 7a). While prebiotic treatment reduced fasting blood glucose level with SMD of −0.10 [95% CI, −0.41, 0.21; *p* = 0.52], acarbose and glipizide increased fasting blood glucose with SMD of 0.15 [95% CI, −0.26, 0.57; *p* = 0.48] and 0.25 [95% CI, −0.33, 0.83; *p* = 0.41], respectively. However, differences between the various treatments (acarbose, glipizide and prebiotic) and the control were not significant (*p* > 0.05). The meta-analysis revealed that the various treatments did not differ significantly (*p* > 0.05) from control (Figure 7b).

Postprandial Blood Glucose.

**Figure 8:**
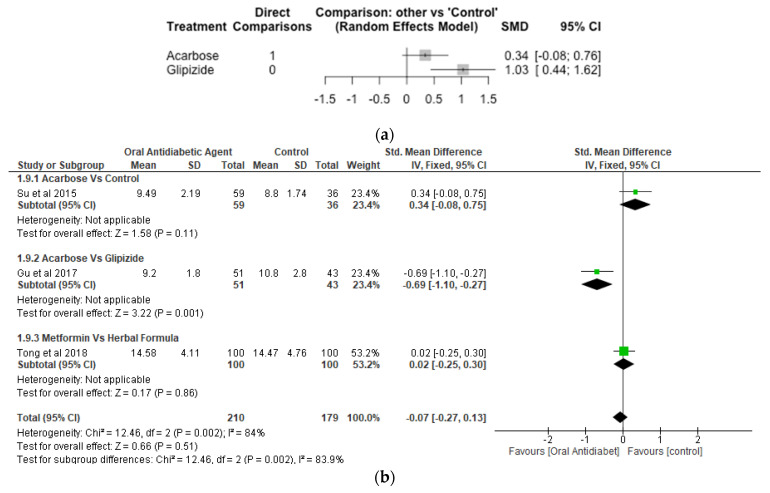
Network Meta-analysis (**a**) and Meta-analysis (**b**) of the effect of treatments versus control on Postprandial Blood Glucose [40,45,46].

Two studies, 189 number of observations and 3 treatments were included in the Network meta-analysis of postprandial blood glucose (Figure 8a). While the difference between acarbose and control were not significant (*p* > 0.05), glipizide increased postprandial blood glucose significantly with SMD of 1.03 [95% CI, 0.44, 1.62; *p* = 0.001]. The result of the meta-analysis revealed that acarbose significantly (*p* < 0.05) reduced postprandial blood glucose compared to gliplizide, while the effect of acarbose compared to control, and metformin compared to herbal formula were not significantly different (*p* > 0.05) (Figure 8b).

## 12. Body Mass Index (BMI)

Soare et al. [43] reported prebiotic significantly reduced BMI compared with control in patients with type 2 diabetes. Similarly, after 3 months of treatment, reductions in body weight and body mass index were more pronounced in the acarbose group than in the Glipizide group [40]. Although BMI was reduced by dapagliflozin, it was increased by gliclazide [47]. Furthermore, *Scutellaria baicalensis* with metformin or placebo did not change the BMI after 8 weeks of treatment [42].

**Figure 9:**
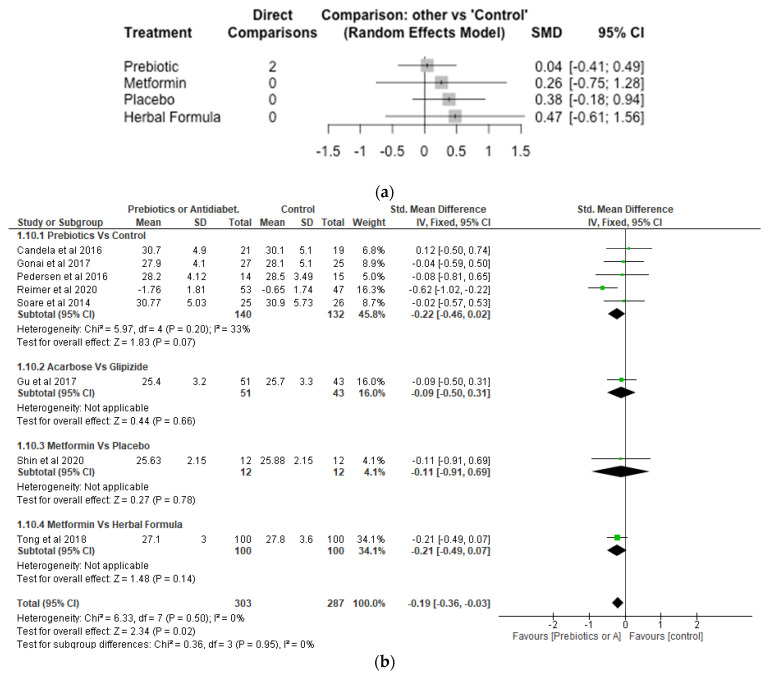
Network Meta-analysis (**a**) and Meta-analysis (**b**) of the effect of treatments versus control on Body Mass Index [32,38,39,40,41,42,43,46].

With respect to the Network meta-analysis, 7 studies, 496 participants and 5 treatments were included (Figure 9a). Although there were increases in BMI in the different treatments (herbal formula, metformin and prebiotic) compared with control, the differences were not significant (*p* > 0.05). The SMD was 0.04 [95% CI, −0.41, 0.49; *p* = 0.86] for prebiotic, 0.26 [95% CI, −0.75, 1.28; *p* = 0.61] for metformin and 0.47 [−0.61; 1.56; *p* = 0.39] for herbal formula, respectively, compared with control. The result of the meta-analysis showed the effects of prebiotics, acarbose and metformin were not significantly different (*p* > 0.05) from control with respect to BMI (Figure 9b).

## 13. Discussion

The results of the Network meta-analysis demonstrated that prebiotics significantly reduced (*p* < 0.05) HbA1c in patients with T2D compared to control. In addition, anti-diabetic agents including glipizide and metformin also reduced HbA1c, although these did not differ significantly (*p* > 0.05) compared to control.

While prebiotics increased the relative abundance of *Bifidobacterium* and *Akkermansia*, it did not differ significantly (*p* > 0.05) compared to control. On the other hand, metformin decreased the relative abundance of *Bifidobacterium*, but increased *Lactobacillus* and *Akkermansia*, although these did not differ significantly (*p* > 0.05) compared with control.

With respect to fasting blood glucose and BMI, the effects of prebiotics and oral antidiabetic agents did not differ significantly (*p* > 0.05) from controls.

The findings of this Network meta-analysis confirm the earlier results of the systematic review and meta-analysis carried out by Zhang et al. [21], Mahboobi et al. [21] and Wang et al. [22] which demonstrated prebiotics were effective in reducing glycated haemoglobin in patients with T2D. However, these earlier reviews did not include gut microbiota as one of the outcomes measured. Fallucca et al. [7] found microbiotic Ma-Pi 2 diet which is rich in carbohydrates, whole grains and vegetables significantly improved glycated haemoglobin in patients with T2D. It was reported that the diet could modulate the composition of gut microbiome [7].

According to Mahboobi et al. [21] the underlying mechanisms of action of prebiotics are based on the fact soluble fibres can delay gastric emptying, slow down glucose entry into the blood stream and reduce the rise of postprandial blood glucose. Furthermore, soluble fibres may alter the production of glucagon like peptide-1 (GLP-1) which is a gut hormone involved in the metabolism of glucose [21]. Soluble fibres may also lead to the production of SCFAs which may influence serum glucose and insulin levels [21]. With respect to glucose lowering agents, the mechanism of action on gut microbiome may relate to their role in lowering inflammatory cytokines and promoting production of SCFAs [14].

Patients with T2D have been shown to exhibit intestinal dysbiosis [31]. Decreases in *Bifidobacterium*, *Roseburia*, *Faecalibacterium* and *Akkermansia* have been associated with T2D [19,49,50]. Ghorbani et al. [19] reported that *Bifidobacterium* is inversely associated with T2D and that the role of *Lactobacillus* appears to be species dependent. For example, *Lactobacillus acidophilus* and *Lactobacillus salivarius* species positively correlated with T2D, while *Lactobacillus amylovorus* species are negatively associated with T2D [19]. *Akkermansia muciniphila* is reported to have a role in the homeostasis of glucose and in protecting against insulin resistance and T2D [19].

Diets high in fat such as Western diets may cause gut microbiota dysbiosis which can lead to increased levels of lipopolysaccharide, oxidative stress, pro-inflammatory cytokines, gut inflammation, gut permeability and insulin resistance [2,49].

Therefore, dietary intervention with prebiotics can substantially modulate gut and faecal microbiota through increases in alpha diversity and regulating the relative abundance of specific bacteria species, independent of antidiabetic drugs [31,37,38].

According to Ghorbani et al. [19], prebiotics are non-digestible fibres which can be fermented by the gut microbiome and can promote the growth of some bacteria. Prebiotic carbohydrates are composed mainly of inulin, fructo-oligosaccharide and galacto-oligosaccharides which are resistant to digestion in the small intestine [51]. However, they are fermented in the large intestine and have been reported to promote the abundance of *Bifidobacterium* and/or *Lactobacillus* [51]. Prebiotics promote eubiosis and attenuates pathological changes of dysbiosis, leading to promotion in the abundance of *Lactobacillus*, *Bifidobacterium*, *Faecalibacterium* and *Bacteroidetes* [49]. Other changes due to the effects of prebiotics include decreases in lipopolysaccharides, oxidative stress, proinflammatory cytokines and gut permeability, and improvements in gut motility and insulin sensitivity [49]. Prebiotics also promote GLP–1 and peptide YY [2]. Supplementation with prebiotics has been shown to improve appetite control of human subjects [2].

The SCFAs including propionate, butyrate, and acetate which are produced from the fermentation of complex carbohydrates including prebiotics are responsible for initiating the various metabolic pathways which regulate glycaemic control and inflammation [19,49]. Acetate has been reported to regulate appetite both directly and indirectly and can stimulate the production of GLP-1 and peptide YY which are appetite suppressing hormones from the L-cells of the intestine [19]. GLP-1 is an insulinotropic hormone which can regulate glucose homeostasis [19]. Propionate can also stimulate the production of GLP-1 and peptide YY, while propionate and butyrate can inhibit pro-inflammatory cytokines [19]. Butyrate, is useful in modulating intestinal barrier permeability and in ensuring pro-inflammatory products do not gain access from the lumen of the gut to the internal milieu [51]. This is important as it has been reported that the translocation of lipopolysaccharide promotes pro- inflammatory cytokines, low grade systemic inflammation, impairs glucose metabolism and increases insulin resistance and T2D [19].

Therefore, in order to promote an increase in the abundance of beneficial bacteria and ensure effective glycaemic control, it is essential that the type and amount of prebiotics consumed and the duration are considered [32]. For example, long term adherence to high fibre plant based diet and daily supplement with inulin type fructans have been reported to be effective in modulating gut microbiota and regulating glycaemic control [31,37]. Furthermore, combining different functional foods may modify human microbial community and improve glycaemic control [31].

Metformin has been reported to promote the growth of SCFA producing microbial species including *Bifidobacterium bifidum* and *Bifidobacterium adolescentis* and increased abundance of *Akkermansia muciniphila* and down regulating *Clostridia* [52]. The primary hypoglycemic effect of metformin is its role in inhibiting hepatic gluconeogenesis [52]. Gu et al. [40] reported acarbose impedes the breakdown and absorption of carbohydrates in the small intestine, and these provide the substrate for microbial fermentation in the large intestine and therefore promotes the abundance of saccharolytic bacteria such as *Lactobacillus* and *Bifidobacterium* species.

## 14. Limitations

The few studies available and the small sample sizes of some of the studies limit the power of this Network meta-analysis to detect statistical differences. While the current findings provide a foundation for assessing the relative effects of the different treatments, our results should be considered exploratory and that further studies are needed to fully examine the effects of prebiotics and oral anti-diabetic agents on gut microbiome and glycaemic control in patients with T2D.

## 15. Conclusions

The results of this systematic review and Network meta-analysis showed prebiotics were significantly (*p* < 0.05) more effective in reducing HbA1c than control in patients with T2D. However, the effects of prebiotics and oral antidiabetic agents did not differ significantly (*p* > 0.05) from the controls with respect to fasting blood glucose, post-prandial blood glucose, body mass index and the genera of gut bacteria examined.

More studies are required to fully investigate the effects of prebiotics and oral antidiabetic agents in patients with type 2 diabetes.

## Figures and Tables

**Table 1:** Search Terms Based on PICOS Tool.

Patient/Population	Intervention	Outcome (Primary)	Study Designs	Combining Search Terms
Patients with diabetes	Prebiotics OR Oral anti-diabetic agents	Gut microbiome	Randomised controlled trial	
Diabetes mellitus, type 2 OR Diabetes complications OR Patients with diabetes OR diabetes mellitus OR type 2 diabetes OR Diabetes	Prebiotics OR Dietary fibre OR Fibre OR Polysaccharide OR Dietary carbohydrate OR Resistant Starch OR carbohydrate OR Oral anti-diabetic agents OR metformin or gliclazide OR acarbose	Microbiome OR Gastrointestinal microbiota OR Gut microbiota OR Microbiota	#1 Randomized OR Randomised controlled trial OR placebo OR controlled clinical trial OR therapy OR randomly OR drug OR trial OR groups #2 “Animals” NOT “Humans” #3 #1 NOT #2	1st Column + 2nd Column + 3rd Column + 4th Column

Abbreviation/Symbol: # (Number).

**Table 2:** Description and characteristics of studies included.

Citation/Country of Study	Type of Study	Aim	Participants	Sample Size	Mean Age (Years)	Mean Diabetes Duration (Years)	Interventions	Results/Findings
Arias-Córdova et al. [36] Mexico	RCT	To assess the effects of NBS and HMS on GC and GV in patients with T2D when treatments were matched for digestible starch content.	All participants with T2D treated with metformin or a combination of glibenclamide and metformin	*n* = 10	48.5 ± 9.12	Not Applicable	NBS, HMS and DMS. including three treatment phases, each with a duration of 4 days, and washout period between treatments of 9-day.	The intake of NBS showed a reduction in fasting glycemia compared to DMS.
Birkeland et al. [37] Norway	RCT	To examine the effect of inulin-type fructans on faecal microbiota and SCFAs in patients with T2D.	T2D, with 2/3 of participants receiving glucose-lowering drugs	*n* = 25		4.7 (0.2–20.0)	16 g Inulin-type fructans versus 16 g maltodextrin. There was 4-week washout which separated the 6 weeks treatment period.	There was moderate, but significant increase in faecal levels of bifidobacteria in the group supplemented daily with inulin-type fructans.
Candela et al. [38] Italy	RCT	To explore the effect of microbiotic Ma-Pi 2 diet in modulating gut microbiota dysbiosis in patients with T2D.	Patients with T2D	Ma-Pi 2 diet: *n* = 21 Control diet: *n* = 19	66	Not Applicable	Fibre rich microbiotic Ma-Pi 2 diet is enriched with complex carbohydrates, legumes, fermented products, sea salt and green tea.	FBG and PBG were reduced significantly in both Ma-Pi 2 and control diets, although this was significantly higher in the Ma-Pi 2 diet compared to control. Both diets were also effective in supporting the recovery of health promoting SCFA producing bacteria including *Faecalibacterium*, *Roseburia*, *Bacteroides* and *Akkermansia*. Increases in *Collinsella* and *Streptococcus* were only counteracted by Ma-Pi 2 diet.
Gonai et al. [39] Japan	RCT	To explore the effects of GOS on on glycaemia, gut microbiota and metabolitic parameters in patients with T2D.	Patients with T2D	GOS group: 28 Placebo group: 27	GOS group: 55 ± 11 Placebo group: 54 ± 12	GOS group: 10 ± 8 Placebo group: 6 ± 5	10 g/d GOS syrup versus 10 g/d maltodextrin syrup. 4 weeks of treatment.	After consumption of GOS, Bifidobacteriaceae was significantly restored in patients with T2D, whereas lipopolysaccharide binding protein and glucose tolerance did not show improvement.
Gu et al. [40] China	RCT	To compare the effect of Acarbose versus sulfonylurea Glipizide on metabolic parameters, (e.g., glycaemic, control plasma BAs), and the intestinal microbiota, and discriminate such changes from disease-dependent alterations.	Patients with T2D	Acarbose group: 51 Glipizide group: 43	Acarbose group: 53 ± 7 Glipizide group: 54 ± 7	Not Applicable	Acarbose treatment versus Glipizide treatment. A 3-month treatment period.	Both the acarbose and glipizide groups improved glycemic control, with no significant differences. Acarbose increased the relative abundances of Lactobacillus and Bifidobacterium and depleted Bacteroides. However, Glipizide treatment did not affect the relative abundances at species-level. After 3 months of treatment, reductions in BW and BMI were more significant in the Acarbose group compared to the Glipizide group.
Medina-Vera et al. [31] Mexico	RCT	To assess functional food-based dietary intervention on biochemical parameters and faecal microbiota in patients with T2D.	Patients with T2D	DP group: *n* = 28 Placebo group: *n* = 25	DP group: 50.4 ± 8.7 Placebo group: 49.8 ± 10.6	DP group: 4.1 ± 3.5 Placebo group: 4.4 ± 3.9	A dietary portfolio, DP (14 g of dehydrated nopal, 4 g of chia seeds, 30 g of soy protein and 4 g of inulin) versus placebo (28 g of calcium caseinate and 15 g of maltodextrin). The treatment period was for 3 months.	Consumption of DP promoted the abundance of Bifidobacterium longum which has been reported to improve insulin sensitivity. There was significant reduction in the levels of HbA1c in patients with T2D in the DP group.
Pedersen et al. [32]	RCT	To investigate the effects of prebiotic supplementation on intestinal bacteria in patients with type 2 diabetes	Patients with type 2 diabetes	Prebiotic group: *n* = 14 Placebo group: *n*= 15	Prebiotic group (56.7 ± 6.0) Placebo group (58.1 ± 6.6)	Prebiotic group (4.6 ± 2.2) Placebo group (4.0 ± 3.1)	Prebiotic (galacto-oligosaccharide mixture) or placebo (maltodextrin supplements each given 5.5g/day for 12 weeks.	Prebiotic fibre supplementation did not improve glucose control or abundance of bacteria compared with control.
Reimer et al. [41] Canada	RCT	To assess the effect of the soluble viscous fibre PGX on glycemic control in adults withT2D.	T2D patients	PGX group: *n* = 147; Placebo group: *n* = 143	PGX group: 56.2 ± 8.6 Placebo group: 53.4 ± 9.9	Not Applicable	PGX (15–20 g/day) versus placebo (rice flour, 6.4–8.6 g/day) 52 weeks of treatment	PGX group increased Roseburia and led to a sustained reduction in HbA1c and FBG compared to placebo.
Shin et al. [42] Korea	RCT	To investigate whether the combination of SB and metformin influenced T2D symptoms.	T2D on 500 mg/day metformin	*n* = 12	SB + Metformin: 63.1 Placebo: 63.1	Not Applicable	SB (3.52 g SB extract) + metformin versus placebo+ metformin. A 4-week washout separated the 8 weeks of treatment	Lactobacillus and Akkermansia, showed significant increases after SB + metformin treatment. The glucose, HbA1c and BMI were not changed after 8 weeks of SB and placebo treatment.
Soare et al. [43] Italy	RCT	To compare the effects of the Ma-Pi 2 diet and the dietary guidelines for T2D recommended by professional societies in Italy on T2D patients.	Overweight or obese (BMI:27–45 kg/m^2^), aged 40–75 years affected by T2D	Ma-Pi 2 diet:25 Control diet:26	Ma-Pi 2 diet: 67 ± 8.16 Control diet: 65 ± 7.28	Ma-Pi 2 diet:7 ± 7.793 Control diet:4.5 ± 8.845	Fibre-rich Ma-Pi 2 macrobiotic diet versus recommended diet of type 2 diabetes by professional societies. 3 weeks of treatment.	The patients that received Ma-Pi 2 diet showed significant reduction in FBG, PBG, HbA1c, and BMI compared to those receiving the recommended diet for T2D.
Soare et al. [44] Italy	RCT	To investigate whether the benefits of Ma-Pi 2 extended beyond the 21-day intensive dietary intervention.	Overweight or obese (BMI 27–45 kg/m^2^), aged 40–75 years affected by T2D.		Ma-Pi 4 diet: 65 ± 8.89 Control diet:64 ± 8.15	Ma-Pi 4 diet: 7 ± 7.41 Control diet: 4 ± 6.67	Fibre-rich Ma-Pi 4 macrobiotic diet versus recommended diet of T2D diabetes by professional societies. 6 months of treatment.	The Ma-Pi 4 diet had great improvement in glycemic control, compared with the control group. Body weight loss was also observed in Ma-Pi 4 group, but was not significantly different compared to the control group.
Su et al. [45] China	RCT	To evaluate the effects of acarbose add-on therapy on gut microbiota and inflammatory cytokines among Chinese patients with T2D.	Patients with T2D that did not receive acarbose for at least 1 month.	Acarbose group: 59 Control group: 36	Acarbose group: 55.7 ± 11.0 Control group: 56.5 ± 10.2	Not Applicable	50 mg acarbose (t.i.d) a day with meals together with oral antidiabetic drugs and/or insulin or insulin analogs versus similar antidiabetic treatment to interventional group but without acarbose. Four weeks of treatment.	Treatment with acarbose can increase the abundance of Bifidobacterium longum in patients with T2D and improve glycemic control.
Tong et al. [46] China	RCT	To evaluate the role of gut microbiota during improvements in hyperglycemia and hyperlipidemia by two drugs: metformin and AMC for diabetic patients with hyperlipidemia.	Patients with T2D and Hyperlipidemia.	Metformin group: 100 AMC group: 100	Metformin group: 58.55 ± 9.17 AMC group: 59.00 ± 9.46	Not Applicable	AMC twice daily versus metformin tablets 0.25 g/time and 3 times/day. 12 weeks of treatment.	The effect of AMC in regulating the microbes in the gut and in improving HOMA-IR and triglyceride levels was more profound compared with metformin.
van Bommel et al. [47] Netherlands	RCT	To examine the effects of 12-week treatment with the SGLT2 inhibitor dapagliflozin and sulphonylurea gliclazide on gut microbiome composition in patients with T2D treated with metformin.	All participants with T2D treated with metformin as monotherapy	Dapagliflozin group: *n* = 24; Gliclazide group: *n* = 17.	Dapagliflozin group: 63 ± 7 Gliclazide group: 63 ± 7	Dapagliflozingroup: 9.8 ± 4.1 Gliclazide group: 10.7 ± 7.3	10 mg dapagliflozin and 30 mg gliclazide. Treatment for 12 weeks.	Both dapagliflozin and gliclazide reduced HbA1c and FBG, while BMI was reduced by dapagliflozin, but increased by gliclazide.
Wu et al. [18] Spain	RCT	To investigate the effect of metformin on the composition and function of the microbiota.	Individuals with type 2 diabetes	Metformin group: *n* = 22 Placebo group: *n* = 18	Metformin group: 52.6 ± 9.4 Placebo group: 54.9 ± 8.1	Not applicable	A start dose of 425 mg/day of metformin and increased progressively to reach 1700 mg/day or placebo (calorie restricted diet). Treatment was for four months.	Metformin and not calorie restricted diet had significant effect on composition and function of the gut microbiota and reduction in HbA1c and fasting blood glucose levels.
Zhao et al. [48] China	RCT	To examine the effect of dietary fibre on SCFA-producing strains in patients with type 2 diabetes.	Individuals with type 2 diabetes	High fibre diet group: *n* = 27 Control group: *n* = 16	High fibre diet group: 58.4 ± 32.2 Control group: 59.7 ± 24.0	High fibre diet group: 8.0 ± 30.1 Control group: 7.9 ± 20	High fibre diet composed of whole grains, traditional Chinese medicinal foods, and prebiotics.	The presence of SCFA producers in greater diversity and abundance by fibre. Participants had better improvement in HbA1c levels.

Abbreviations: BMI: Body Mass Index; T2D—type 2 diabetes; Ma-Pi —macrobiotic diet; FBG—fasting blood glucose; PBG—post-prandial blood glucose; HbA1c—glycated haemoglobin; GOS—galacto-oligosaccharides; AMC—an herbal formula consisting of eight herbs; HOMA-IR—homeostatic model assessment of insulin resistance; DP—dietary portfolio; SB—scutellaria baicalensis; SCFA—short chain fatty acids; PGX—PolyGlycopleX, a highly viscous polysaccharide complex; NBS—native banana starch; HMS—high-amylose maize starch; DMS—digestible maize starch; GC—glycemic control; GV—glycemic variability; BA—bile acid; BW—body weight; SGLT2—sodium-glucose-linked transporter-2.

**Table 3:** Effects of prebiotics and Oral antidiabetic agents on gut microbiome.

Studies	Bifidobacterium	Lactobacillus	Roseburia	Bacteroides	Ruminococcus	Clostridium	Akkermansia
Birkeland et al. [37] Norway	There was moderate, but significant increase in faecal levels of bifidobacteria in the group supplemented daily with inulin-type fructans.	N/A	N/A	*Bacteroides ovatus* was enriched by the prebiotic fibre	N/A	N/A	N/A
Candela et al. [38] Italy	N/A	N/A	Ma-Pi 2 diet and control were effective in supporting the recovery of *Roseburia*	Ma-Pi 2 diet and control were effective in supporting the recovery of *Bacteroides*	Ma-Pi 2 diet and control supported the reduction of *Ruminococcus*	N/A	Ma-Pi 2 diet and control resulted in the increase of *Akkermansia*
Gonai et al. [39] Japan	*Bifidobacterium* was significantly restored after consumption of GOS	N/A	N/A	N/A	Ruminococcus was significantly lower after consumption of GOS	N/A	N/A
Gu et al. [40] China	The relative abundances of *Bifidobacterium* species increased in Acarbose group.	Acarbose group increased the relative abundances of *Lactobacillus* species.	N/A	The intervention of Acarbose depleted the relative abundances of Bacteroides species.	N/A	Acarbose group depleted the relative abundances of *Clostridium* species.	N/A
Medina-Vera et al. [31] Mexico	Consumption of dietary portfolio stimulated the abundance of *Bifidobacterium longum.*	N/A	N/A	N/A	N/A	N/A	Dietary portfolio increased *Akkermansia muciniphila*
Pedersen et al. [32]	The effect of prebiotic treatment on *Bifidobacterium* was not significant.	The effect of prebiotic treatment on *Lactobacillus* was not significant.	The effect of prebiotic treatment on *Roseburia* was not significant.	N/A	N/A	The effect of prebiotic treatment on *Clostridium* was not significant.	N/A
Reimer et al. [41] Canada	*Bifidobacterium* Spp. changed significantly over time after PGX.	*Lactobacillus* was greater in the placebo compared with the PolyGlycopleX	The relative abundance of *Roseburia* was significantly increased by the soluble viscous fibre PolyGlycopleX	N/A	N/A	*Clostridium coccoides* changed significantly over time after PGX	*Akkermansia muciniphila* changed significantly over time after PGX
Shin et al. [42] Korea	The relative abundance of *Bifidobacterium* was significantly lower in the scutellaria baicalensis and metformin group compared to placebo.	*Scutellaria baicalensis* and metformin increased *Lactobacillus* significantly compared to placebo.	N/A	N/A	N/A	N/A	Scutellaria baicalensis and metformin increased *Akkermansia* significantly compared to placebo.
Su et al. [45] China	Acarbose treatment can increase the content of gut *Bifidobacterium longum* in type 2 diabetes mellitus patients.	N/A	N/A	N/A	N/A	N/A	N/A
Tong et al. [46] China	N/A	N/A	*Roseburia* was enhanced by herbal formula	N/A	N/A	N/A	There was decrease in *Akkermansia* in the metformin treated group
van Bommel et al. [47] Netherlands	N/A	N/A	N/A	N/A	N/A	N/A	*Akkermansia muciniphila* was not significantly affected by Dapagliflozin or Gliclazide treatment.
Wu et al. [18] Spain	There was increase in *Bifidobacterium adolescentis* after metformin treatment	N/A	N/A	N/A	N/A	N/A	N/A

Abbreviations: N/A (Not Applicable).

## Data Availability

Secondary data analysis of publicly available data was carried out.

## Supplementary Materials

The following supporting information can be downloaded at: https://www.mdpi.com/article/10.3390/nu14235139/s1, Figure S1: Network Plots; Table S1: Age & Sex Distribution of Treatments.

## Abbreviations

AMC—herbal formula consisting of eight herbs; BA—bile acid; BMI—body Mass Index; BW—body weight; CI—confidence interval; DMS—digestible maize starch; DP—dietary portfolio; FBG—fasting blood glucose; GC—glycemic control; GLP-1—glucagon like peptide -1; GOS—galacto-oligosaccharides; GV—glycemic variability; HbA1c—glycated haemoglobin; HMS—high-amylose maize starch; HOMA-IR—homeostatic model assessment of insulin resistance; Ma-Pi —macrobiotic diet; N/A: Not Applicable; NBS—native banana starch; NMA—network meta-analysis; PBG—post-prandial blood glucose; PGX—PolyGlycopleX, a highly viscous polysaccharide complex; PICOS—Population, Intervention, Comparator, Outcomes, Studies; PRISMA—Preferred Reporting Items for Systematic Reviews and Meta-Analyses; RCT—randomised controlled trial; SB—scutellaria baicalensis; SCFA—short chain fatty acids; SGLT2—sodium-glucose-linked transporter-2; SMD—standardised mean difference; T2D—type 2 diabetes.
