# Supplementary material for: The Effect of Prebiotics and Oral Anti-Diabetic Agents on Gut Microbiome in Patients with Type 2 Diabetes: A Systematic Review and Network Meta-Analysis of Randomised Controlled Trials
Source: Nutrients. 2022 Dec 2;14(23):5139. doi: 10.3390/nu14235139 (PMC9739188; doi:10.3390/nu14235139)
Supplement: Supplementary file 1 [file nutrients-14-05139-s001.zip › nutrients-2043707-Supplementary Materials.pdf]

Figure S1. Network Plots.

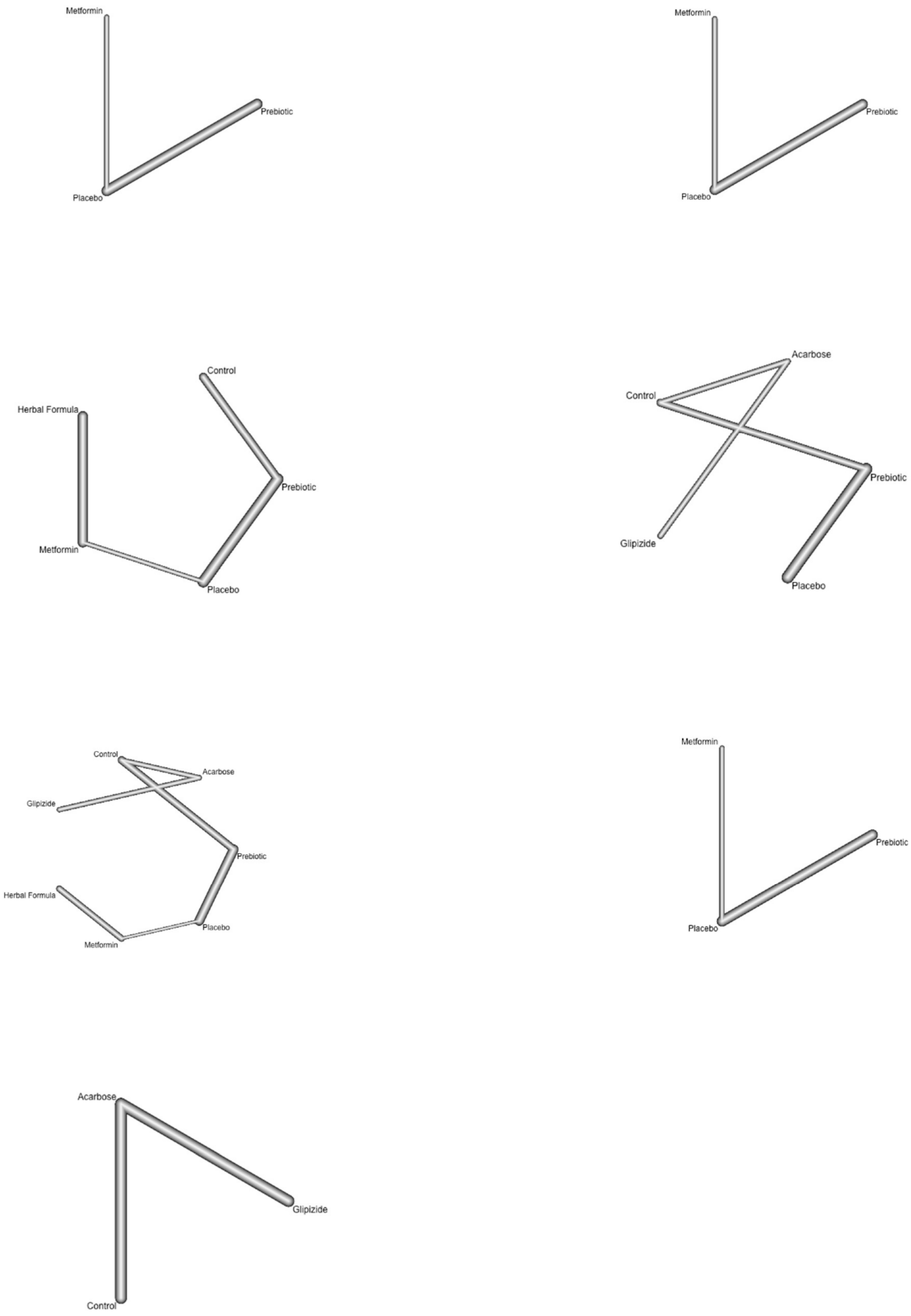

Table S1. The effect of prebiotics and oral anti-diabetic agents on gut microbiome in patients with type 2 diabetes – Mean Age (Years) and Sex Distribution

Outcomes	Treatments	Mean Age (years)	Sex Distribution
Bifidobacterium	Prebiotics Vs Placebo	Prebiotic: 54.4<br>Placebo: 53.8	Prebiotic Male: 29<br>Prebiotic Female: 57<br>Placebo Male: 27<br>Placebo Female: 57
	Metformin Vs Placebo	Metformin: 63.1<br>Placebo: 63.1	Metformin Male: 5<br>Metformin Female:3<br>Placebo Male: 4<br>Placebo Female:5
Lactobaccilus	Prebiotics Vs Placebo	Prebiotic:56.2<br>Placebo:53.4	Prebiotic Male: 50<br>Prebiotic Female:97<br>Placebo Male: 42<br>Placebo Female: 101
	Metformin Vs Placebo	Metformin: 63.1<br>Placebo: 63.1	Metformin Male: 5<br>Metformin Female:3<br>Placebo Male: 4<br>Placebo Female:5
Akkermansia	Prebiotics Vs Placebo	Prebiotic:56.2<br>Placebo:53.4	Prebiotic Male: 50<br>Prebiotic Female:97<br>Placebo Male: 42<br>Placebo Female: 101
	Metformin Vs Placebo	Metformin: 63.1<br>Placebo: 63.1	Metformin Male: 5<br>Metformin Female:3<br>Placebo Male: 4<br>Placebo Female:5
Glycated Haemoglobin	Prebiotics Vs Control	Prebiotic: 58.4<br>Control: 57.7	Prebiotic Male: 17<br>Prebiotic Female: 24<br>Control Male: 16<br>Control Female: 23
	Acarbose Vs Control	Acarbose: 55.7<br>Control: 56.5	Acarbose Male: 32<br>Acarbose Female: 27<br>Control Male: 17<br>Control Female: 19
	Acarbose Vs Glipizide	Acarbose: 53<br>Glipizide: 54	Acarbose Male: 34<br>Acarbose Female: 17<br>Glipizide Male: 24<br>Glipizide Female: 19
	Metformin Vs Herbal Formula	Metformin: 58.55<br>Herbal Formula: 59.00	Metformin Male: 50<br>Metformin Female: 50<br>Herbal Formula Male: 50<br>Herbal Formula Female: 50
	Metformin Vs Placebo	Metformin: 63.1<br>Placebo: 63.1	Metformin Male: 5<br>Metformin Female:3<br>Placebo Male: 4

			Placebo Female:5
Fasting Blood Glucose	Prebiotics Vs Control	Prebiotic: 58.66<br>Control: 58.04	Prebiotic Male: 20<br>Prebiotic Female: 29<br>Control Male: 18<br>Control Female: 28
	Acarbose Vs Control	Acarbose: 55.7<br>Control: 56.5	Acarbose Male: 32<br>Acarbose Female: 27<br>Control Male: 17<br>Control Female: 19
	Acarbose Vs Glipizide	Acarbose: 53<br>Glipizide: 54	Acarbose Male: 34<br>Acarbose Female: 17<br>Glipizide Male: 24<br>Glipizide Female: 19
	Metformin Vs Herbal Formula	Metformin: 58.55<br>Herbal Formula: 59.00	Metformin Male: 50<br>Metformin Female: 50<br>Herbal Formula Male: 50<br>Herbal Formula Female: 50
Postprandial Blood Glucose	Acarbose Vs Control	Acarbose: 55.7<br>Control: 56.5	Acarbose Male: 32<br>Acarbose Female: 27<br>Control Male: 17<br>Control Female: 19
	Acarbose Vs Glipizide	Acarbose: 53<br>Glipizide: 54	Acarbose Male: 34<br>Acarbose Female: 17<br>Glipizide Male: 24<br>Glipizide Female: 19
	Metformin Vs Herbal Formula	Metformin: 58.55<br>Herbal Formula: 59.00	Metformin Male: 50<br>Metformin Female: 50<br>Herbal Formula Male: 50<br>Herbal Formula Female: 50
Body Mass Index	Prebiotics Vs Control	Prebiotic: 58.73<br>Control: 57.63	Prebiotic Male: 23<br>Prebiotic Female: 32<br>Control Male: 21<br>Control Female: 32
	Acarbose Vs Glipizide	Acarbose: 53<br>Glipizide: 54	Acarbose Male: 34<br>Acarbose Female: 17<br>Glipizide Male: 24<br>Glipizide Female: 19
	Metformin Vs Placebo	Metformin: 63.1<br>Placebo: 63.1	Metformin Male: 5<br>Metformin Female:3<br>Placebo Male: 4<br>Placebo Female:5
	Metformin Vs Herbal Formula	Metformin: 58.55<br>Herbal Formula: 59.00	Metformin Male: 50<br>Metformin Female: 50<br>Herbal Formula Male: 50

			Herbal Formula<br>Female: 50
